# Supplementary material for: The COVID-19 Pandemic and Ophthalmic Care: A Qualitative Study of Patients with Neovascular Age-Related Macular Degeneration (nAMD)
Source: Int J Environ Res Public Health. 2022 Aug 2;19(15):9488. doi: 10.3390/ijerph19159488 (PMC9368447; doi:10.3390/ijerph19159488)
Supplement: Supplementary file 1 [file ijerph-19-09488-s001.zip › ijerph-1798250-supplementary.pdf]

Supplementary Table S1. Interview schedule

**Interview schedule**
1. Could you tell me about your experiences of going to the eye clinic for your regular check-ups before March 2020?
2. Have you attended any clinic appointments since March 2020?
3. Could you tell me about your thoughts or experiences of attending clinic appointments since March 2020?
4. Could you tell me about the types of changes the COVID-19 pandemic has had on your eye care?
5. Could you tell me about any changes to appointments since March 2020?
6. Could you tell me about the kinds of social activities you or your family took part in before March 2020?
7. Could you tell me about effects the pandemic has had on your life in general?
8. Have you done anything to help deal with any changes that have happened?
9. Do you have any worries or concerns related to the pandemic?
10. Would you like to add anything or ask any questions?

Supplementary Table S2. Coding framework

	Code	Definition
1	Burden/Perceived amount of effort	The perceived amount of effort that is required to participate in a target behaviour
2	Self-efficacy	The participant's confidence that they can perform a given task; this also includes a lack of confidence
3	Attitude	An individual's evaluative judgement of a target behaviour on some dimension (e.g. good/bad, harmful/beneficial, pleasant/unpleasant, also ambivalence)
4	Behavioural intention	An individual's motivation or willingness to exert effort to perform a target behaviour
5	Actual behaviour/acceptance	The action of undertaking a target behaviour
6	Subjective norm	An individual's perception of the degree to which important other people approve or disapprove of a target behaviour
7	Image	The degree to which an individual perceives that a target behaviour will enhance his or her status in his or her social system
8	Individual differences	Individual difference variables include personality and/or demographics (e.g., traits or states of individuals, gender, and age) that can influence individuals' perceptions of a target behaviour
9	Reported experience	Participant actual experiences
10	General health status	Participant's perception of their general health status
11	Perceived threat	An individual's perception of severity and susceptibility that eye health may deteriorate e.g. AMD no longer treatable, will lose sight or progression of AMD to other eye

12	Health beliefs and concerns about eye health	An individual's beliefs and concerns around AMD
13	Aging	Believes around functional, health, sensory, cognitive and mobility changes
14	Medical Services satisfaction	The extent to which participants are satisfied with current health care services for nAMD
15	Affordability of health services	Refers to the affordability of health services e.g. private health care within NHS context
16	Comfort with health services	Refers to psychological feelings of patients towards health services and hospital environment e.g. cleanliness of hospital
17	Professionalism of healthcare staff	Refers to knowledge, skills and interpersonal skills of healthcare staff
18	Safety of healthcare	Participant's perception of healthcare safety e.g. experienced medical teams, complete medical facilities, hospital security measures
19	Waiting time	Patient's perception of waiting time for appointments, treatment etc.
20	Information quality	Quality of inforamtion provided on care
21	Healthcare professional's (Doctor's) opinion (similar to Subjective norm)	Influence of healthcare professionals as they are perceived to be a point of expert authority
22	Other influencing factors	Participant makes references to factors not otherwise covered by codes within this framework
23	Family support (not Subjective norms)	Participant makes reference to the presence of family or a significant other
24	Emotion	Participant describes stress, anxiety or experience of trauma
25	Major life event	Participant refers to major life event

47	Social context	Participant describes their living space, residential area, whether or not they live with others
26	Experience with eye care	Participant describes what happens or what has happened when they have been in contact with macular, optometry or ophthalmologist service
27	Travel	Participant describes how they get to their usual appointments, includes incurred expenses or parking difficulties
